# Cardiac Hormones Target the Ras-MEK 1/2-ERK 1/2 Kinase Cancer Signaling Pathways

**DOI:** 10.3390/cancers3011182

**Published:** 2011-03-08

**Authors:** David L. Vesely

**Affiliations:** Departments of Internal Medicine, Molecular Pharmacology and Physiology, Cardiac Hormone Center, University of South Florida Health Sciences Center, J. A. Haley Veterans Medical Center-151, 13000 Bruce B. Downs Blvd., Tampa, Florida 33612, USA; E-Mail: david.vesely@va.gov; Tel.: +1-813-972-7624; Fax: +1-813-972-7623

**Keywords:** Ras, MEK 1/2 kinases, ERK 1/2 kinases, DNA synthesis, cancer, signaling

## Abstract

The heart is a sophisticated endocrine gland synthesizing the atrial natriuretic peptide prohormone which contains four peptide hormones, *i.e.*, atrial natriuretic peptide, vessel dilator, kaliuretic peptide and long-acting natriuretic peptide, which decrease up to 97% of human pancreatic, breast, colon, prostate, kidney and ovarian carcinomas as well as small-cell and squamous cell lung cancer cells in cell culture. *In vivo*, these four cardiac hormones eliminate up to 80% of human pancreatic adenocarcinomas, two-thirds of human breast cancers, and up to 86% of human small-cell lung cancers growing in athymic mice. Their signaling in cancer cells includes inhibition of up to 95% of the basal activity of Ras, 98% inhibition of the phosphorylation of the MEK 1/2 kinases and 97% inhibition of the activation of basal activity of the ERK 1/2 kinases mediated via the intracellular messenger cyclic GMP. They also completely block the activity of mitogens such as epidermal growth factor's ability to stimulate ERK and Ras. They do not inhibit the activity of ERK in healthy cells such as human fibroblasts. The final step in their anticancer mechanism of action is that they enter the nucleus as demonstrated by immunocytochemical studies to inhibit DNA synthesis within cancer cells.

## 1. Introduction

The Ras-mitogen-activated protein kinase (MAPK)/extracellular signal-related kinases (ERK) cascade, hereafter referred to as the Ras-MAPK pathway, is a prototypical signal transduction pathway that is aberrantly activated in many neoplasmas including prostate and breast cancers [1,2]. This pathway's activation is associated with a poor prognosis [2]. Structural alteration in the upstream GTPase Ras occurs in 25% to 30% of human cancers, which allows them the ability to relay mitogen signals in a ligand-independent manner, thereby obviating the need for ligand activation of growth factor receptors that occurs in normal cells [3,4]. In addition, this pathway contributes to enhanced survival of tumor cells while also facilitating their metastatic spread to distant organs [3]. The integral role of the Ras-MEK-ERK pathway in mediating multiple hallmarks of cancer has suggested that the different kinases in this pathway may be targets for the treatment of cancer [5,6]. Attempts to target Ras by perturbing its interaction with either Son of Sevenless gene (SOS) or growth factor receptor-bound 2 (GRB2) have not yielded viable drug development candidates largely because of the inherent difficulties of disrupting protein-protein interactions with drug-like molecules [3]. Several drug discovery programs have been devoted to finding inhibitors of farnesyltransferase as a means to prevent the membrane localization of Ras [3]. Despite the successful identification of several chemical leads that effectively inhibited this prenylation enzyme, tumor cells, however, have proved generally to be impervious to the action of this class of inhibitors [3].

**Figure 1.**
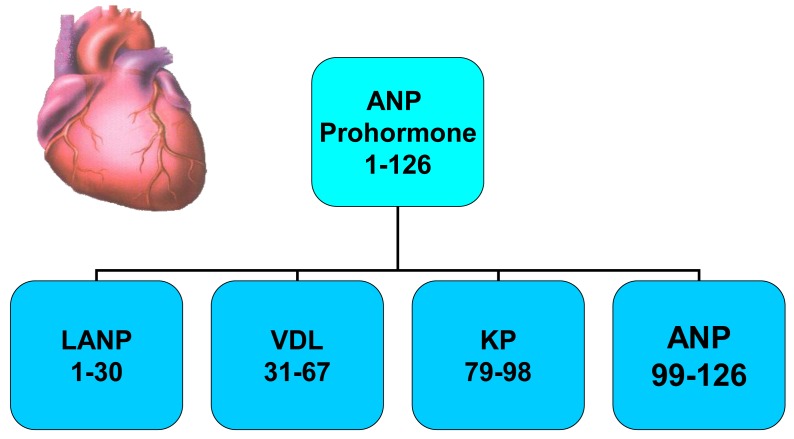
The atrial natriuretic peptide gene in the heart synthesizes a 126 amino acid (a.a.) prohormone with which proteolytic processing results in the formation of four cardiac hormones. These four cardiac hormones, *i.e.*, (**1**) long acting natriuretic peptide (LANP) consists of the first 30 amino acids of the 126 a.a. prohormone; (**2**) vessel dilator (VDL), a.a. 31-67 of the prohormone; (**3**) kaliuretic peptide (KP), a.a. 79-98 of this prohormone and (**4**) atrial natriuretic peptide (ANP), consisting of a.a. 99-126 of the 126 a.a. prohormone. Reprinted with permission from [11].

A novel group of anticancer agents, *i.e.*, peptides made by the human heart (Figure 1) have at least a portion of their anticancer signaling via the Ras-MAPK pathway [7-12]. These peptides are termed atrial natriuretic peptides as they are synthesized mainly in the atrium rather than the ventricles of the heart in healthy animals and humans. They are peptides and have sodium excreting properties (natriuresis) in healthy humans [13,14]. These peptides are hormones as they are synthesized in one organ (heart), circulate throughout the body, and have biologic effects of causing a natriuresis in another organ (kidneys). The atrial natriuretic peptide gene synthesizes a prohormone containing four peptide hormones which are involved in blood pressure regulation and maintenance of plasma volume in animals [15-20] and humans [21-23]. These peptide hormones, numbered by their amino acid (a.a.) sequences beginning at the N-terminal end of the atrial natriuretic peptide (ANP) prohormone, consist of the first 30 a.a. of the prohormone, *i.e.*, long-acting natriuretic peptide (LANP), a.a. 31–67 (*i.e.*, vessel dilator), a.a. 79–98 (kaliuretic peptide) and a.a. 99–126 (Figure 1) [24,25].

## 2. Cardiac Peptides' Effects on Cancer Cells *in Vitro*

The four cardiac hormones from the ANP prohormone decrease up to 97% of human pancreatic, colon, prostate, breast, ovarian and kidney adenocarcinoma cells [26-31], angiosarcoma of the heart cells [32], melanomas [33], medullary thyroid carcinomas [34], glioblastomas of brain [35], as well as small-cell [36] and squamous cell lung carcinoma cells [37] in cell culture within 24 hours. There was a 97.4%, 87%, 88% and 89% (p < 0.001 for each) decrease of human prostate adenocarcinoma cells secondary to vessel dilator, long-acting natriuretic peptide, kaliuretic peptide, and atrial natriuretic peptide, respectively, within 24 hours at their 1 mM concentrations, without any proliferation in the three days following this decrease [28].

## 3. Cardiac Peptides Eliminate Human Cancers Growing in Athymic Mice

### 3.1. Pancreatic Adenocarcinomas

The five-year survival rate of persons with adenocarcinoma of the pancreas is 1% with a median survival of only four months [38,39]. Current cancer chemotherapy and surgery prolong survival by a few months, but the mean survival is four months for persons treated with surgery and/or current cancer chemotherapeutic agents [38,39].

When each of these peptides at 3 nM min^-1^ kg^-1^ body weight were infused subcutaneously for 28 days in athymic mice bearing human pancreatic adenocarcinomas, ANP eliminated 80% the human pancreatic cancers [40]. Vessel dilator, LANP and kaliuretic peptide eliminated the primary pancreatic cancers in 33%, 20%, and 14% of their respective treatment groups [40]. In those animals in which the pancreatic adenocarcinomas were eliminated in the primary site, not a single animal ever had a recurrence in the primary site [40]. One ANP-treated animal developed a metastatic lesion and this lesion was eliminated with treatment with vessel dilator [40]. Even in the treated animals which did not have total elimination of their human pancreatic adenocarcinoma with the osmotic infusion pumps changed weekly, their tumor volume decreased to less than 10% (and with vessel dilator to less than 2%) of that of the untreated animals both during treatment and in a 12-month follow-up period [40].

### 3.2. Breast Cancer

Vessel dilator, LANP, kaliuretic peptide and ANP eliminate 67%, 50%, 67% and 33% of the human breast adenocarcinomas in athymic mice when infused subcutaneously for 28 days with weekly fresh hormones at 3 nM min^-1^ kg^-1^ body weight [41]. There was no recurrence of the breast cancers in the primary site and no metastasis except in the ANP-treated group [41]. The natriuretic peptide receptors −A and −C were decreased 50% and 31%, respectively, in metastatic *versus* primary ANP-treated breast adenocarcinomas, as a possible reason why less of the breast cancers responded to ANP compared to the other three cardiac hormones as ANP works via these decreased receptors while the other peptide hormones have their own specific receptors [14,41].

### 3.3. Small-cell Cancer of the Lung

LANP, vessel dilator, kaliuretic peptide, ANP and urodilatin (a peptide made in the kidney by differential processing of the ANP prohormone, which consists of ANP plus the four C-terminal amino acids of kaliuretic peptide attached to it) eliminate 86%, 71%, 57%, 43% (p < 0.001 for the cardiac hormones) and 25% (p < 0.05; urodilatin) of the human small-cell lung carcinomas [42]. The treated small-cell lung carcinomas that were not eliminated grew rapidly, similar to the untreated controls, whose volume was 7-fold larger in one week, 18-fold increased in two weeks, 39-fold increased in three weeks, 63-fold increased in one month and 97-fold increased in volume in six weeks [42]. One vessel dilator treated small-cell lung carcinoma animal developed a large tumor (8,428 mm^3^ volume) on treatment and this tumor was eliminated utilizing ANP and then LANP sequentially, each for four weeks [42]. Table 1 summarizes the ability of each of the four cardiac hormones to eliminate human breast, pancreatic and small-cell lung cancers growing in athymic mice.

## 4. Cardiac Peptides' Signaling Targets in Cancer Cells

### 4.1. Ras

**Figure 2.**
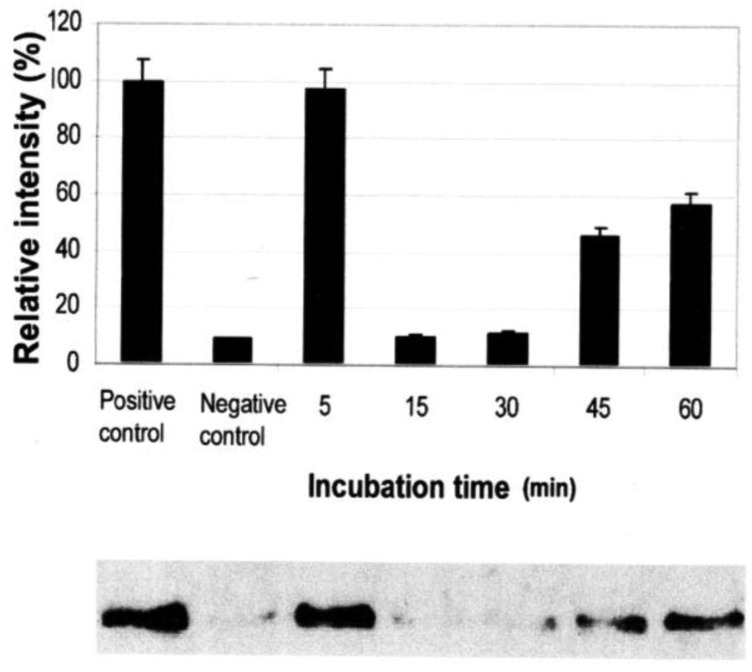
Dose response of vessel dilator on the activation of Ras in human prostate cancer cells at 0.1 μmol/L in time-sequenced experiments at 5, 15, 30, 45, and 60 minutes. There was a significant (p < 0.0001) inhibition of the activation of Ras at each time point where evaluated by analysis of variance (ANOVA). Ras-GPT (*i.e.*, active Ras) is at 21 kD. The relative intensity in these bar graphs is a comparison of three Western blots against the positive control (untreated Ras-GPT) with one typical Western blot illustrated. The illustrated negative control in this graphs is Ras-GDP at 21 kDa. Reprinted with permission from [7].

Vessel dilator and kaliuretic peptide (each 1 μM) inhibit the activation of Ras GTP from inactive Ras GDP by 95% (p < 0.0001) (Figure 2) and 90% (p < 0.0001), respectively [7]. At 0.01 μM of kaliuretic peptide, the maximal inhibition was 95%. The inhibition of Ras lasted for 48 to 72 hours secondary to both peptides [7]. Their ability to inhibit Ras was inhibited by cyclic GMP antibody and cyclic GMP itself inhibited Ras phosphorylation (89%; p = 0.0015) [7].

Atrial natriuretic peptide (ANP) and long-acting natriuretic peptide (LANP) reduced the activation of Ras-GTP over a concentration range of 0.01 μM to 1 μM [8]. ANP and LANP (each 0.1 μM) inhibited the activation of Ras by 90% (*p* < 0.0001) and 83% (*p* < 0.0001), respectively [8]. At 0.01 μM of LANP, the maximal inhibition was 89%, which occurred within 5 minutes. Both peptide hormones inhibited Ras for three to four hours [8]. Their ability to inhibit Ras was inhibited by cyclic GMP antibody and cyclic GMP itself inhibited Ras phosphorylation (72%; p = 0.009) [8]. Thus, atrial natriuretic peptide, vessel dilator, kaliuretic peptide and long-acting natriuretic peptide inhibit Ras at least partially mediated via cyclic GMP as part of their anticancer mechanism(s) of action [8].

### 4.2. MEK 1/2 Kinases

The prototype member of the MEK kinase family, designated MAP kinase kinase (MKK-1)/or MEK-1, specifically phosphorylates the MAP kinase regulatory threonine and tyrosine residues present in the Thr-Glu-Tyr motif of ERK 1/2 [43,44]. A second MEK family member, *i.e.*, MEK-2, resembles MEK-1 in terms of its substrate specificity but is seven residues longer than MEK-1 with the amino acid sequence of MEK-2 being 81% identical to MEK-1 [43].

**Figure 3.**
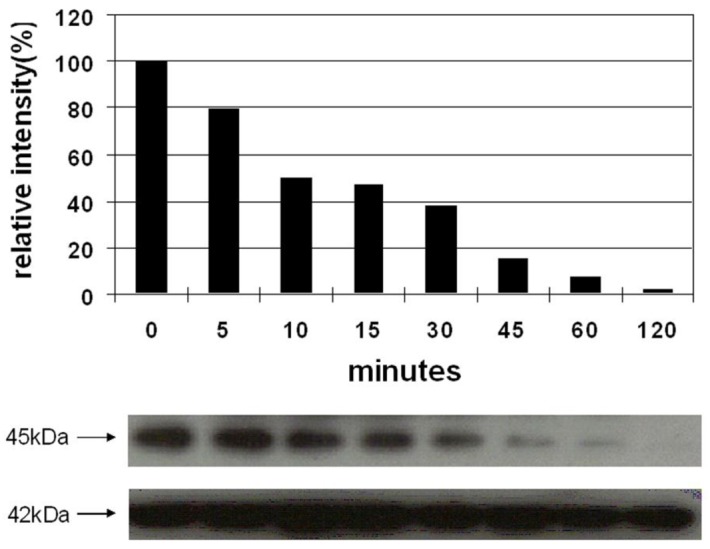
Vessel dilator at 10 μM inhibits 98% of the phosphorylation of mitogen-activated protein kinase kinase (MEK 1/2), which was maximal at two hours and significant at p < 0.00001 when evaluated by analysis of variance (ANOVA). MEK 1/2 is at 45 kDa while B-actin (loading control) is 42 kDa. The relative intensity in the bar graphs is a comparison against untreated MEK 1/2 (100% intensity). Reprinted with permission from [9].

Vessel dilator and kaliuretic peptide decrease the activation of MEK 1/2 over a concentration range of 0.01 μM to 10 μM [9]. Vessel dilator and kaliuretic peptide (each 10 μM) inhibited the phosphorylation of MEK 1/2 kinase by 98% (p < 0.0001) (Figure 3) and 81% (p < 0.001), respectively [9]. The inhibition of MEK 1/2 lasted for at least two hours, where it was maximal, secondary to both peptides [9]. Their ability to inhibit MEK 1/2 was inhibited by cyclic GMP antibody and cyclic GMP itself inhibited MEK 1/2 phosphorylation, suggesting that cyclic GMP was important for mediating these cardiac hormones' effects [9].

ANP and LANP decreased the activation of MEK 1/2 over a concentration range of 0.01 μM to 10 μM [10]. LANP and ANP (each 10 μM) inhibited the phosphorylation of MEK 1/2 kinase by 97% (p < 0.00001) and 88% (p < 0.00001), respectively [10]. The inhibition of MEK 1/2 was maximal at two hours and ceased by four hours secondary to both peptides [10]. The ability of peptides to inhibit MEK 1/2 was inhibited by cyclic GMP antibody and cyclic GMP itself inhibited MEK 1/2 phosphorylation by 93% [10]. Thus, ANP, vessel dilator, kaliuretic peptide and LANP each inhibit MEK 1/2 kinase mediated via cyclic GMP as part of their anticancer mechanism(s) of action [9,10].

### 4.3. ERK 1/2 Kinases

Extracellular-signal regulated kinase (ERK) 1/2 is a mitogen activated protein kinase (MAP kinase) important for the growth of cancer(s) [45,46]. Growth factors such as epidermal growth factor (EGF), fibroblast growth factor, platelet derived growth factor and vascular endothelial growth factor (VEGF), after binding to their specific receptor tyrosine kinases, work via ERK 1/2 kinase to cause proliferation [45]. EGF, for example, when it binds to its EGF receptor, causes this receptor to autophosphorylate on tyrosine residues and recruits the Grb2-Sos complex to turn on membrane-associated Ras, which then activates the Ras/Raf-Mek 1/2-ERK 1/2 kinase cascade [45]. Of the mitogen-activated protein kinases, ERK 1 and 2, 42 and 44 kDa proteins, can directly translocate to the nucleus and stimulate DNA synthesis and the production of several intermediate early genes such as c-fos and c-myc, which are implicated in causing cells to divide and grow [45,46].

Vessel dilator and kaliuretic peptide decrease the phosphorylation of ERK 1/2 kinases over a concentration range of 0.01 μM to 1 μM [11]. Vessel dilator and kaliuretic peptide (each 1 μM) inhibit the phosphorylation of ERK 1/2 kinase by 96% and 70% (p < 0.001), respectively [11]. Both have significant effects within five minutes at a concentration of 0.01 μM [11]. The inhibition of ERK 1/2 lasted for at least two hours secondary to both [11]. Their ability to inhibit ERK 1/2 was inhibited by cyclic GMP antibody and cyclic GMP itself inhibited ERK 1/2 phosphorylation [11], suggesting that cyclic GMP is important for mediating their mechanisms of action [11]. Vessel dilator and kaliuretic peptide both inhibit ERK 1/2 kinase mediated via cyclic GMP as a third metabolic target in their anticancer mechanism(s) of action.

**Figure 4.**
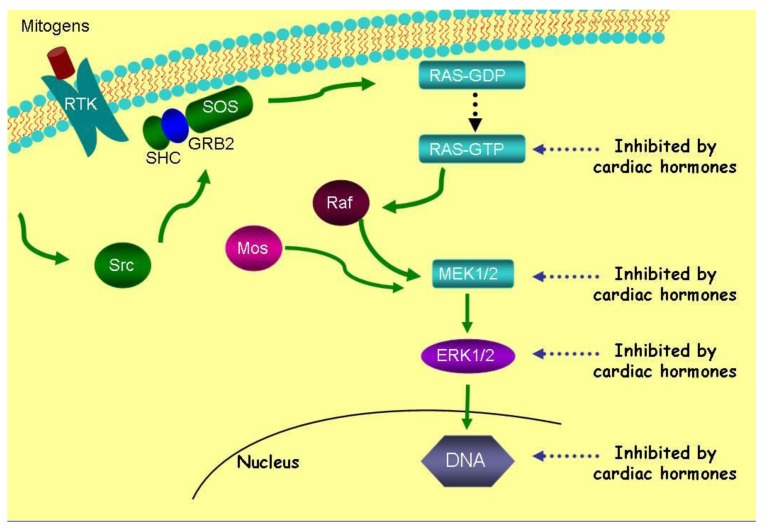
Cardiac hormones inhibit five metabolic targets, *i.e.*, Ras-GTP, MEK 1/2, and ERK 1/2 kinases of the Ras-MEK 1/2-ERK 1/2 kinase cascade by 95-98%. They are also strong inhibitors (*i.e.*, 91%) of DNA synthesis within cancer cells. Reprinted with permission from [54].

ANP and LANP, likewise, decrease the activation of ERK 1/2 kinases over a concentration range of 0.01 μM to 10 μM [12]. ANP and LANP's maximal inhibition of the phosphorylation of ERK 1/2 kinases were 94% and 88% (p < 0.0001), respectively [12]. ANP had significant effects within five minutes at a concentration of 10 μM. The inhibition of ERK 1/2 kinases lasted for at least two hours, where it was maximal, secondary to ANP and LANP. Their ability to inhibit ERK 1/2 was inhibited by cyclic GMP antibody and cyclic GMP itself inhibited ERK 1/2 phosphorylation, suggesting that cyclic GMP mediates their effects of inhibition the phosphorylation of ERK 1/2 kinases [12]. Thus, the cardiac hormones inhibit five metabolic targets, *i.e.*, Ras-GTP, MEK 1/2 kinases and ERK 1/2 kinases as illustrated in Figure 4.

### 4.4. Mitogens Stimulation of ERK 1/2 Kinases and Ras Blocked by Cardiac Peptides

#### 4.4.1. ERK 1/2 Kinases

Growth promoting hormones such as epidermal growth factor (EGF) and insulin work as mitogens via ERK 1/2 (mitogen-activated) protein kinase (MAP kinase) to cause growth [45,46].

Insulin (1 μM) and EGF (10 ng/mL) each enhance the phosphorylation of ERK 1/2 by 66% [47]. This enhanced phosphorylation of ERK 1/2 by EGF and insulin was decreased to 10%, 8%, 27% and 13% above non-stimulated ERK 1/2 by vessel dilator, kaliuretic peptide, LANP and ANP [47].

#### 4.4.2. Ras

Insulin's ability to contribute to cancer formation and proliferation is thought to be mediated in part by its ability to convert inactive GDP-Ras to active GTP-Ras [48]. Vessel dilator, LANP, ANP and kaliuretic peptide, each at 1 μM, inhibit 88%, 94%, 56% and 47%, respectively, of insulin's (1 μM) activation of Ras [49]. Likewise, epidermal growth factor (EGF) has been shown to directly activate Ras [50-53]. Vessel dilator, LANP, ANP and kaliuretic peptide, each at 1 μM, inhibit 73%, 79%, 33% and 45%, respectively, of 5 ng/mL EGF stimulation of Ras [54].

### 4.5. Cardiac Hormones do not Inhibit ERK 1/2 Kinase in Normal Human Fibroblasts

Vessel dilator and kaliuretic peptide do not inhibit ERK 1/2 in normal human fibroblasts in a concentration range of 10 pM (physiologic) to 100 nM (0.1 μM) as they do in cancer cells [55]. Likewise, the cardiac hormones inhibit proliferation of viable human pancreatic cells but at the same concentrations there is no decrease in proliferation of human prostate, kidney, lung or endothelial cells compared with untreated prostate, kidney, lung or endothelial cells [56].

### 4.6. DNA Synthesis

All four of these cardiac hormones synthesized by the ANP gene localize to the nucleus of human pancreatic adenocarcinomas by immunocytochemical evaluation [57,58], where they can inhibit DNA synthesis.

Vessel dilator, LANP, kaliuretic peptide and ANP, each at 1 μM concentration, inhibit DNA synthesis when incubated with pancreatic adenocarcinoma cells for 24 hours by 91%, 84%, 86% and 83%, respectively (P < 0.001 for each) [26]. One of the known mediators [59,60] of these peptide hormones' mechanism(s) of action, *i.e.*, cyclic GMP, inhibited DNA synthesis in these adenocarcinoma cells by 51% [26]. Dose-response curves revealed that 8-bromo-cyclic GMP, the cell permeable analog of cyclic GMP, decreased DNA synthesis in these cancer cells 46%, 42%, 39%, and 34% (all P < 0.05) at concentrations of 3 mM, 1 mM, 100 μM, and 1 μM, respectively [26]. Even at 1 nM (*i.e.*, 10^−9^ M) of 8-bromo-cyclic GMP there was a 25% decrease in DNA synthesis in the adenocarcinoma cells (P < 0.05) [26]. At 100 pM of 8-bromo cyclic GMP, its effects on DNA synthesis in these adenocarcinoma cells became not significant (14% decrease) [26].

## 5. Conclusions

Vessel dilator, LANP, kaliuretic peptide and ANP inhibit DNA synthesis 80–91% in all human cancer cell lines [26-37]. Thus, after inhibiting ERK 1/2, DNA synthesis (a further or final step in the Ras-MEK 1/2-ERK 1/2 kinase pathway) is inhibited within the nucleus (Figure 4). These peptide hormones' ability to inhibit DNA synthesis is specifically mediated by the intracellular mediator cyclic GMP as when a cyclic GMP antibody is incubated with the cardiac hormones they are unable to inhibit DNA synthesis [29]. Thus, with respect to signaling in cancer cells, after inhibiting the Ras-MEK 1/2-ERK 1/2 kinase cascade (Figure 4), the cardiac peptides can inhibit DNA synthesis secondarily by ERK 1/2 kinases' mediated decrease in DNA synthesis or possibly directly since they have been shown by immunocytochemical studies [57,58] to enter the nucleus where they can inhibit DNA synthesis.

## Figures and Tables

**Table 1.** Cardiac Hormones Ability to Eliminate Human Cancer Growing in Athymic Mice.

	**Breast Cancer**	**Pancreatic Adenocarcinoma**	**Small-cell Lung Cancer**
**VDL**	67%	33%	71%
**LANP**	50%	20%	86%
**ANP**	33%	80%	43%
**KP**	67%	14%	57%

The numbers in each column are the percentages of human cancers which are eliminated and never recur in the primary site in athymic mice when treated with each of the cardiac hormones for 28 days at 3 nM/kg body weight/minute. Abbreviations: VDL = vessel dilator, LANP = long acting natriuretic peptide, ANP = atrial natriuretic peptide, and KP = kaliuretic peptide.
